# Barriers to seeking healthcare services and contributing factors to grade 2 disability among women affected by leprosy in Telangana, India – a qualitative study

**DOI:** 10.1186/s12939-025-02642-9

**Published:** 2025-09-29

**Authors:** Charlotte Nehring, Andrea Kaifie, Ananth Reddy, Matthew Willis, Fabian Schlumberger, Nita Chaudhuri, Anil Fastenau

**Affiliations:** 1 Institute for Occupational, Social, and Environmental Medicine, Friedrich-Alexander University Erlangen-Nürnberg, Erlangen, Germany; 2 Sivananda Rehabilitation Home, Hyderabad, India; 3 Department of Global Health, Institute of Public Health and Nursing Research, University of Bremen, Bremen, Germany; 4 Marie Adelaide Leprosy Centre, Karachi, Pakistan; 5 Faculty of Health, Medicine and Life Sciences, Maastricht University, Maastricht, The Netherlands; 6 German Leprosy and Tuberculosis Relief Association (DAHW), Wuerzburg, Germany

## Abstract

**Background:**

Leprosy, a neglected tropical disease, remains a significant global health issue, with India accounting for nearly 60% of cases in 2022. Untreated Leprosy can result in irreversible disabilities and lead to social stigma, significantly affecting the lives of patients and their families. This study explores the barriers faced by women with leprosy in accessing healthcare and other factors that contributed to the development of Grade 2 disability in India.

**Methods:**

Qualitative data were gathered through 20 interviews with women affected by leprosy at the Sivananda Rehabilitation Home, a leprosy clinic in Hyderabad, India. An interview guide was developed to conduct semi-structured interviews, specifically regarding the time between the onset of symptoms, diagnosis, and treatment start. An inductive analysis followed by the application of Levesque et al.’s framework was undertaken to identify themes and patterns in the participants’ experiences with the disease and treatment.

**Results:**

Six key themes were identified. The social environment plays a pivotal role in disease progression, with participants often prioritising societal expectations over their own health, such as being good wives and mothers. Stigmatisation led to social isolation, as many women avoided contact outside their families to hide deformities. Most participants visited several healthcare facilities before receiving a diagnosis, facing financial and emotional burdens. Communication gaps were evident both within healthcare facilities - where companions were sometimes informed before the patient – and in their social environments. Finally, individual factors such as lack of knowledge, awareness, and trust in medical advice also contributed to care-seeking delays.

**Conclusions:**

This study highlights significant gaps in healthcare access for women with leprosy in India. Family dynamics, societal roles, and stigma delay care, while physical and emotional burdens add to challenges. Communication gaps and limited awareness further reinforce neglect and mistrust. Addressing these barriers is crucial for effective policy and program implementation to reduce the burden of leprosy among women.

**Supplementary Information:**

The online version contains supplementary material available at 10.1186/s12939-025-02642-9.

## Introduction

Leprosy, or Hansen’s disease, is classified as a neglected tropical disease (NTD). Over a billion people globally are affected by NTDs and are underrepresented in global health agendas. Leprosy, like other NTDs, is linked to low socio-economic status and poor living conditions, severely affecting individuals and their families. Disease consequences include financial strain, disability, stigma, social exclusion, and discrimination. Caused by *Mycobacterium leprae*, leprosy is a chronic infectious disease likely spread through droplets during prolonged contact, with symptoms typically emerging after a long incubation period of over five years [1]. They include hypopigmented or erythematous skin patches. As the disease progresses, it can damage peripheral nerves, causing loss of sensitivity and irreversible disability, often due to delayed or improper treatment [2]. In endemic countries, leprosy is usually diagnosed through altered skin areas, thickened peripheral nerves, or microscopic detection of bacteria in a slit-skin smear [3]. Treatment involves multidrug therapy (MDT) for six to twelve months and is provided free of charge worldwide [3]. The disease is curable, but existing disabilities and nerve damage cannot be reversed and require ongoing care [4].

The World Health Organisation (WHO) has established a grading system for leprosy patients that indicates their level of disability and the interval between symptom onset and diagnosis [5]. Grade 0 represents normal sensation with no visible impairments; Grade 1 indicates loss of protective sensitivity without visible impairments, and Grade 2 includes visible impairments or disabilities. Later stages of the disease can lead to visible impairments from nerve damage, such as absorption, burns, scars, ulcers, and weakness with contractures, classified as Grade 2 disability (G2D) [5, 6]. The prevalence of leprosy patients with G2D is associated with late diagnosis and a high disease burden, indicating the current state of disease elimination efforts [7]. Treating the disease not only prevents disability in those affected but also stops transmission and represents an important component of the WHO strategy 2021–2030, “Towards zero leprosy” [1]. This strategy aims to reduce the rate of new cases of G2D by 90% per million inhabitants [1, 8, 9]. According to the WHO, more than 174,000 Leprosy cases were reported globally in 2022, with nearly 60% occurring in India [10].

Overall, the global number of leprosy patients in the last decade has decreased, including in India. However, India still faces significant challenges in combating the disease [11]. The proportion of new leprosy patients with G2D at diagnosis in India increased by 38%, from 1.3% in 2020 to 1.8% in 2022 [10] suggesting longer delays in seeking healthcare and/or inadequate disease management. India, a multi-ethnic nation with over 1.4 billion people, is classified as a lower-middle-income country [12]. Disparities based on caste, gender, and region contribute to persistent poverty, with nearly 15% of the population experiencing multidimensional poverty that affects health, education, and living standards [13]. Women face greater challenges, resulting in poorer health outcomes compared to men [14]. Despite some improvements, significant progress toward gender equality is still needed [14]. Throughout their lives, women experience discrimination and harmful practices like female feticide, infanticide, dowry, and various forms of violence. These factors negatively impact their education and health, contributing to higher unemployment rates and increased gender disparities [14]. They also bear additional burdens from leprosy-related disabilities and societal stigma [15, 16]. Sociocultural, financial, and structural factors affect healthcare access for women in India [17, 18]. Delays in seeking help and diagnosing leprosy pose significant challenges to reducing transmission and the disease’s burden [16].

Leprosy and other NTDs disproportionately affect marginalised groups, including minorities and women [19, 20]. In developing countries, women face reduced access to health services and less healthcare funding than men [21, 22]. Evidence also shows that women are diagnosed less frequently and later than men [23]. Price identified in her review barriers to women’s healthcare access in five countries, including India, such as societal stigma, economic reliance on men, lower social status, and the gender insensitivity of leprosy control programs [16]. Data shows that specific groups, including women, are underrepresented in the number of leprosy cases detected in almost every country worldwide although leprosy can affect both men and women equally [16]. However, the proportion of officially registered Leprosy cases in women in 2022 was globally 38.9%, and in India 38.6% [10]. This highlights the need for more research into the experience of leprosy, particularly in women, to explain the gender disparities in case detection [16].

Currently, little is known about the specific individual and systemic barriers that women with G2D affected by leprosy face in seeking healthcare services in India. Although the study focuses on women in Telangana primarily because the clinic serving these participants is located there, it can be assumed that the findings reflect broader patterns of gendered stigma, institutional neglect, and care-seeking barriers that are prevalent across different regions. Understanding health behaviours and gender differences is crucial to identifying and addressing women’s healthcare obstacles. This study aims to address barriers to accessing healthcare services and other factors contributing to the progression of the disease and the development of deformities in women in India. Furthermore, it will explore different factors leading to the progression of the disease and the development of deformities to stage G2D to better understand the phenomenon of delayed diagnosis and disability development, as well as understand the lived experiences from research participants. This study employs an intersectional feminist lens to investigate how the overlapping of gender, female identity, and leprosy shapes the experiences of those affected by leprosy and their access to medical care. The research aims to answer the following research question: What are the perceptions and barriers of women affected by Leprosy to seeking healthcare services in Telangana, a state in India, and what other factors contribute to the progression of their condition to Grade 2 disability?

## Methodology

### Study design

A qualitative descriptive study was conducted to explore and understand the factors contributing to disabilities in women affected by leprosy. It incorporates phenomenological sensitivity to explore the lived experiences. Interviews were utilised to gain deeper insights into the women’s perceptions, uncovering new insights into the complexities surrounding delayed diagnoses. The study aimed to highlight these delays from the women’s perspective, providing an opportunity to glean knowledge from their experiences.

### Study setting

The research took place at the Sivananda Rehabilitation Home (SRH) offices in Hyderabad, south-central India, with support from the German Leprosy Relief Association (GLRA). The clinic handles leprosy detection, diagnosis, and treatment. It offers MDT, physiotherapy, and reconstructive surgery to manage and prevent patient issues, such as nerve damage complications, with some former patients residing there [24, 25].

### Study population

The study involved 20 women affected by leprosy and G2D, either in treatment or post-treatment. Participants were selected to ensure diversity in age, education, and living situation. Due to time and logistical limits, only women residing in or visiting SRH for appointments or check-ups were included. Participant recruitment was ended when data saturation was achieved.

### Selecting participants

Participants were selected from SRH’s database, with eligibility criteria described in Table 1. To align participants with the study’s goals, one author (AR) carried out purposive sampling and another author (CN) confirmed the eligibility. Recruitment was stopped once 20 participants from diverse age groups, educational levels, and cultural backgrounds were interviewed, and the target number was achieved. The profile of study participants is illustrated in Table 2.

**Table 1:** Inclusion and exclusion criteria for research participants

Inclusion criteria	Exclusion criteria
- Female - Diagnosed with leprosy - G2D - Over 18 years old	- Not able to travel to the SRH for the interviews - Other medical conditions, such as mental health problems, which can restrict their interview participation

**Table 2:** Profile of study participants

Code	Age	Marital status	Children	Education	Place of origin, Distance to Hyderabad
P1	28	U	0	< 4 years	City, 30 km
P2	19	U	0	Finished school	City, Hyderabad
P3	25	U	0	Finished school	City, 200 km
P4	28	M	2	Finished school	City, Hyderabad
P5	30	M	3	Unschooled	City, Hyderabad
P6	20	U	0	Finished school	City, 150 km
P7	21	U	0	Unschooled	Village, 150 km
P8	40	S	2	Unschooled	Village, 200 km
P9	27	M	2	Finished school	Village, 80 km
P10	45	W	2	Unschooled	City, Hyderabad
P11	55	U	0	Unschooled	Village, 390 km
P12	38	M	0	Unschooled	Village, 180 km
P13	30	M	1	< 4 years (3rd class)	City, 400 km
P14	32	M	2	< 4 years (2nd class)	Village, about 300 km
P15	49	M	2	< 4 years (2nd class)	City, Hyderabad
P16	50	M	2	< 4 years (2nd class)	Village, 200 km
P17	30	M	0	Unschooled	City, 300 km
P18	50	S	2	Unschooled	Village, 150 km
P 19	75	S	0	4 years	Village, half-day trip
P20	57	S	1	Unschooled	Village, 200 km

*M *Married, *S* Separated from husband, *U* Unmarried, *W* Widow

### Data collection methods

The interviews were conducted by CN, a female doctor and Global Health researcher from Germany who is fluent in English. During her five-week stay from May to June 2024 at the study site, she made efforts to build trust and rapport with participants to ensure the collection of rich data.

A local translator, who was a healthcare worker at the clinic, was present during the interviews to ensure the translation from Telugu into English and vice versa. The translator had long-standing experience in leprosy care and was trusted by the participants, which helped open communication. Prior to the interviews, CN introduced the translator to the basics of qualitative research and the importance of precise translation.

Semi-structured interviews were undertaken using a prepared guide that CN produced with consultation from SRH (Appendix 1) to steer conversations, while allowing participants to share openly, and were on average 40 min long. Questions were adjusted based on initial responses to foster a more natural discussion. In-person interviews included only the participant, translator, and CN, ensuring privacy and comfort given the topic’s sensitive nature. Non-verbal cues, such as body language, contributed to enhanced understanding. With participants’ consent, sessions were audio-recorded for detailed transcription and analysis, with a translator fluent in Telugu and English ensuring seamless communication accuracy.

### Data analysis

CN began data analysis during fieldwork, allowing adjustments to the interview guide as new themes emerged. Some questions were revised or removed. Transcript quotes were edited for clarity; direct quotes were rewritten in the first person to enhance reader connection, with filler words and errors removed. Thematic analysis followed Braun and Clarke’s method [28], using coding schemes to identify themes from the interviews. Atlas.ti was used in the coding process, with quotes supporting findings to ensure objectivity. To further situate the findings within broader contexts, Levesque et al.’s framework was applied as a secondary interpretive lens after the primary inductive thematic analysis. Quotes from participants are marked with “P” for participants. The AI-based tool DeepL was used to improve grammatical accuracy and sentence structure during the writing process.

### Ethical considerations

Ethical clearance was obtained first from the local institutional ethics committee of Ramdevrau’s Hospital (ECR/709/Inst/TG/2015/RR-21) and Maastricht University (FHML/GH_2024.021). Subsequently, participants were informed about the study, its risks, and their right to withdraw. Those who agreed signed a consent form in English or Telugu (Appendices 2 & 3). Illiterate participants had a witness sign on their behalf and provided a fingerprint after verbal consent facilitated by the translator. Consent for audio recording was also obtained. Interviews were recorded and transcribed anonymously for confidentiality; data safety was prioritised. As a token of appreciation for their time and participation, the study participants were offered food and drinks during the interviews. The study was conducted in accordance with the Declaration of Helsinki.

## Results

### Participants demographic profile

Twenty women affected by leprosy with G2D were interviewed. All had taken MDT, with some also undergoing surgery or physiotherapy. Eight participants lived in SRH, while others were inpatients or attending follow-ups at the clinic. Six main themes were identified from the data (Table 3).

**Table 3:** Main and subthemes of data analysis

Main themes	Subthemes	Codes
Social context	Living situation Decision making Delay Protection Neglect Family dynamics	Husband, Society, Friends, Help, Childhood experience, Parents, Alcohol
Women’s role	Daughter Wife Motherhood	Children, Household, Chores, Husband, Parents, Feeding, Cooking, Cleaning, Pregnancy
Stigma	Social isolation Avoidant behaviour Misbeliefs Interpretation of symptoms	Eating alone, Family functions, Appearance, Witchcraft, Village, Religion
Health status	Symptoms Restrictions Emotional state	Fear, Crying, Guilt, Happiness, Shock, Concerns about the future, Suicidal thoughts, Vomiting, Weakness, Loss of sensation, Amputation, Blisters, Infection, Ulcer, Deformities, Footdrop, Patches, Scars, Side-effects, Eat independently, Interrupting education
Visits at healthcare facilities	Several Institutions Journey Treatment Financial Burden Lack of Communication	Frustration, Number of visits, Traditional medicine, Treatment, Sivananda, Money, Bill, Pension, Follow-up, Footwear, Physiotherapy, Surgery, Medication, Public Transport, Husband, Parents, Symptoms, Diagnosis, Treatment
Individual factors	Care-seeking behaviour Lack of knowledge Lack of trust Awareness gap	Employment status, Education, Illiteracy, Lack of Knowledge, Lack of Trust in medical advice, Unawareness

**Fig. 1:**
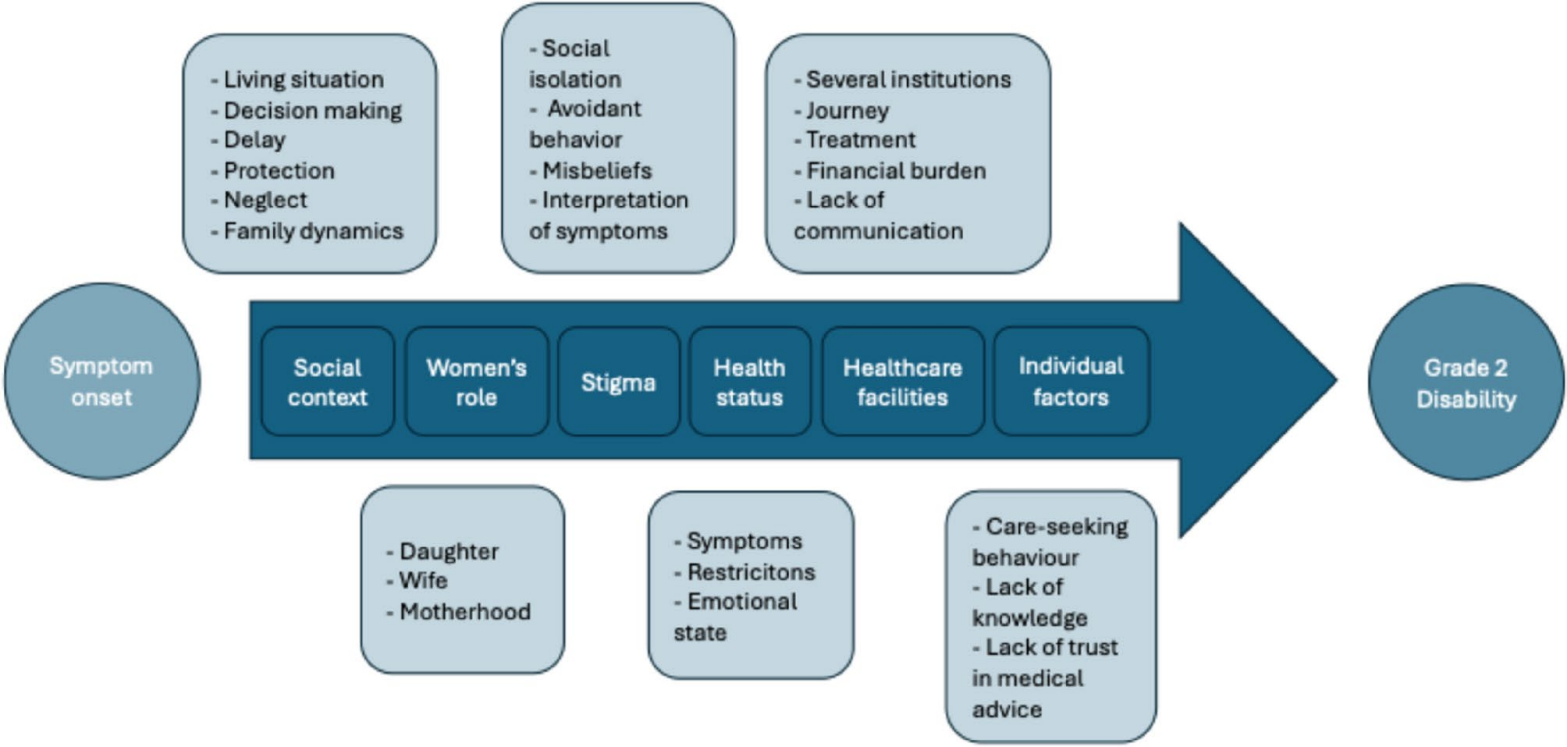
A Conceptual Framework for Healthcare Access Barriers

The six main themes and their interactions within a modified version of Levesque et al.’s framework [27, 28] are also visualised in Figure 1. The model shows the dimensions of healthcare access and the consequence of the identified themes on women’s health-seeking behaviour, resulting in later diagnosis and a higher incidence of G2D. The components within the arrow represent the pathway women take to access healthcare, while the elements outside the arrow indicate the themes identified in the analysis of the interviews.

### Theme 1: social context

#### Living situation and the influence on decisions

During the interviews, all participants highlighted the significant role their social environment played in accessing healthcare, which subsequently affected the progression of their disease. They described how family dynamics and social influences shaped their decisions and basic needs. Both support and neglect from their social surroundings heavily impacted their futures. Most participants did not share their symptoms or diagnosis beyond their household. They explained that decisions about seeking healthcare were often made by their husbands, parents, or siblings, who were involved in both discussing symptoms and choosing when and where to get help. For example, one woman recalled, “First, I shared to mother, then father. (…) we went for hospital and treatment together” (P3). Another noted, “I never expressed to anybody that I am suffering with leprosy, only parents know” (P3). In some cases, husbands decided whether to seek care - “He [husband] decided to go to the hospital, what about this, what is the problem? You have been getting these patches and all” (P4) – while others described family members suggesting alternative medicine: “And then father and sisters said, better to take that tree leave” (P6).

#### Delay

Delays in accessing healthcare services for the first time varied significantly among the women, ranging from a few days to “three months” (P7), and in some cases, to several years. As one participant recalled, “After seeing the patches, after 2-3 days, I went to the hospital” (P2).

For some participants with little to no social support, and who were still children when symptoms appeared, the delay in seeking help lasted years. They recalled growing up without a supportive family, with their leprosy being discovered through accredited social health activists (ASHA) or government-led leprosy campaigns in villages. One participant shared that, despite having deformities and a foot ulcer for years, no one in her family took her for treatment, and it was only when nuns visited the village to detect leprosy that her condition was identified. “At that time, leprosy workers used to go from house to house to detect leprosy. Like that, also the sisters visited my house. (…) So, the sisters brought me here [Sivananda] and also decided that I have to stay here” (P11). Another participant was detected by an ASHA worker when she had already developed deformities. “Nobody took interest in taking me to the hospital. (…) At that time, leprosy workers used to go from house to house to detect leprosy” (P11).

#### Protection

The attitude of people surrounding the participants was decisive for them. Some participants received support and understanding and described the protective behaviour of their families, which helped them to deal with their situation. For instance, one participant shared how her sister’s encouragement prevented her from giving up: “I said to my sister, I want to die, I want to end my life. But sister says, better not to think like that. (…) And also, I stayed with her” (P8). Another reflected on the contrast between family support and social exclusion, noting, “Even though others were separating me from themselves, my family was with me, so that was good” (P15). Despite the support of being accompanied by her mother, one participant expressed her fear related to going to the hospital, stating: “I am afraid to consult a doctor – being a lady” (P7).

#### Neglect

Other participants did not experience support from their surroundings and had to deal with difficult living situations in addition to their health problems. One woman, who lost her parents at a very young age and had to perform hard physical labour during childhood, recounted a traumatic experience related to her illness. She described how, after developing leprosy symptoms, she was stigmatised by her community and even faced an attempt on her life by a family member: “So, when I was very small, at that time I lost my parents, right, so the others like my grandparents (…) used to make me work. So mostly it was chopping the wood (…). During that time my skin got hurt. And then, that wood caused infection, you know. It was untreated, it used to get infected. (…) One of those elderly ladies [in the village] she observed, and she told everyone that I am having a big disease called leprosy and do not touch me, do not talk to me (…). So, from that time onwards my family members, they stopped talking to me, they just pushed me aside. (…) I do not know how to swim. My grandfather told me he is going to wash me and then pushed me into the water and left me there, but somebody else, saw me as a poor child getting drowned, so he pulled me out and saved me” (P15).

Other participants also reported a lack of family support, growing up in households where fathers drank regularly or where the presence of multiple siblings led to a limited capacity for care. For example, one participant shared that their “Father is not that much of a caretaker, because he uses to drink.” (P6) while another stated, “My father did not take care of me. I have nine siblings, so there is no time for him to take proper care“ (P10). Another participant who did not share her problems with anyone, sought help on her own and recalled that “He [her husband] is not taking me, and he does not know. (…) He only sometimes came home, and he never took care of me” (P10).

#### Family dynamics

Some participants reported that their husbands left them after discovering their symptoms or leprosy diagnosis, abandoning them and marrying other women instead of facing the situation together. One participant mentioned that “Because of the patches and ulcer on my feet he left me” (P20), another one was telling: “No divorce, but due to the disease, I came here [to Sivananda] and he married another girl” (P8).

### Theme 2: women’s role 

Participants described various roles and responsibilities based on their living situation. One unmarried women living with her parents spoke of traditional daughterly duties and daily household chores: “I am in the house, whatever work is required, I used to prepare food, cleaning, I am bringing water” (P7). Married participants with children expressed concerns about the burden of being wives, mothers, and managing their disability, which limit their ability to fulfill tasks. One participant reports, “I suffer, I feel very bad, due to this disease and deformities and marriage, children, and husband. I am thinking more” (P5).

Despite knowing the consequences, one participant's housework obligations led to deformities, with her loss of sensation causing repeated, unavoidable injuries. She says: “I was told how to take care, but I could not, because there is work at home, cleaning, washing clothes, cooking, and all those things” (P13).

Motherhood was a common theme in the interviews. Many mothers worried about their children’s well-being and struggled to seek help for themselves while caring for their children. One participant shares “I worry about the children. My hand is like this, how to look after the children, they should be safe. That’s why I kept them at my sister’s house. I am worried more about children and my deformity” (P8). Another participant explains, “I have to leave the children also, so I have to find somebody to look after the children, that was also very difficult. (…) When I went to the hospital, I could not work, so I could not earn money to feed the children” (P10).

The participants' identification as mothers and wives is prioritised over their own health. One participant recalls. “When I came to Sivananda, they advised for admission for ulcer and deformities in my hand, but my husband said due to children I cannot stay here. I didn’t stay at that time, I went home. (…) Husband told, better not to get admission, because children are there at home, to look after children” (P9).

Children and families did not only represent a barrier to healthcare services for some, but they were also directly impacted by the participants’ deformities. One participant discussed her role as a mother, which she must fulfil before she can seek help. She explains, “Women’s problem is more, compared to men. We have got to do things, then only us. How to do it, small kids are there (…). Because due to this problem, I am not looking after the children in a good way, I always think that. Because I am not well. I am not able to feed them properly. I am not able to dress them properly. I worry about that. Before I got this, I did go to the park, (…) and play. We play together. I used to go to the school with my kids. (…) I feel very sad. I am not able to take children properly, I am not able to lift them” (P4).Another participant delayed visiting a doctor due to her pregnancy and her role as a wife and future mother. She recalls, “Because that time I was pregnant, I was afraid to go to the doctor, to take medicine and all. That’s why I never went to the doctor” (P9).

### Theme 3: stigma

Another key theme in many interviews was the stigma participants faced due to the social consequences of leprosy.

#### Social isolation

Participants were treated differently after their diagnosis, leading to social isolation, loneliness, and insecurity. One participant describes, “Before I was diagnosed, everybody was together, we were a happy family, joined family, but then after I was diagnosed with this, then they started separating themselves” (P19). Another shares, “They said that I should be separated, my plate, my glass, everything should be separated – isolated, isolated” (P8). One participant explains, “Parents also never say to anyone that I am suffering with leprosy, because of social stigma” (P7).

Participants faced stigma not only due to leprosy but also because of deformities and disabilities. One women reflects, “Some of the relatives and neighbours (…) they say that they don’t mingle with me. When I am going there, their faces are completely changed. (…) Why am I coming to their house like that? (…) They don’t know I have leprosy, but by seeing my deformities in the hand and ulcer, when I go and move, their attitude has changed” (P9).

Following the advice of other affected patients and their experiences with stigma, one participant decided to move to Sivananda. She recalls, “So, I was taking the medicine and going back home. But then, some patients were here, they told me, I cannot live outside with my deformities, I stay here, and they got me married with one person here” (P14).

One participant faced the disease and its consequences alone, saying, “I had nobody to share my feelings, there is nobody to hear me, I had to process it on my own, and the sadness that is there, I have to face it on my own and keep it within me” (P19).

#### Avoidant behaviour

Out of fear, some participants avoided social interactions with family and friends. One participant shares, “So, on my own, I felt bad, you know, when there was a family occasion like marriage, you have to sit and eat. And at that time, because of my deformities, I would have problems. So, I myself avoided, but the others did not say anything to me” (P16).

Furthermore, the medications' side effects, like temporary skin darkening, also led participants to avoid social contact. One participant explains, “While taking the medicine, I felt weakness, then colour changed, black colour, (…) for 2 years face is completely black. When I became black, I never attended any functions, stayed at home” (P9).

Many female patients indicated that fear of stigma shaped their decision to attend healthcare facilities. One participant, who lied to her neighbours about her visits, explains how she copes with the situation: “If someone knows, in the bus, when I am traveling, some of known persons could be there. By seeing my foot maybe, what are they thinking? (…) Being afraid of that, I always put socks, shoes, and socks. I use to cover that ulcer. (…) Some injury, due to that only, I put bandage” (P9).

Another interviewee feared discrimination from other patients at the healthcare facility, so she tried to visit as infrequently as possible. She recalls, “By seeing me, they might think that I am suffering with leprosy or something. They could be thinking or saying go away from this hospital (…). I used to think that” (P7).

#### Misbeliefs

Leprosy was put in context with misbeliefs. One participant mentioned, “Neighbours told me that my grandparents had it, that is why I got the disease. They did not tell me that it is leprosy, but grandparents had it, so witchcraft is there” (P13).

Some patients felt nothing could help them; they did not trust the medicine or surgeries the healthcare workers offered. This left them feeling helpless, resigned to their fate. One participant stated, “I accepted the fact, that leprosy patients will get this kind of deformities” (P14).

One woman described her situation at home, as her husband thought erroneously that she could transmit the disease to her children via sharing food, even though she had started the treatment at that time already: “Husband is telling, when I am feeding, first I have to feed the children, then I take (…) That is why I feed my children first then only I will go for my meal. Husband is telling for our children’s security only. (…) I should obey, that is good” (P9).

#### Interpretation of symptoms

Participants understood and interpreted the symptoms in their own ways. Some mentioned the reasons and explanations for the changes as they understood them. One participant suggested, “Maybe some effect from some religion – in villages, there are certain rituals, which do cause some kind of difference in the skin” (P20). Another participant recalled, “I used to sleep outside under the tree. Some insects are crawling, and it is that patches appear. First, I thought of that” (P2). A third participant connected the symptoms to a recent childbirth, saying, “I thought, ok, that is due to childbirth, I have given birth to my last child, that time, my health condition is not good, due to that, there is weakness” (P5).

### Theme 4: health status

#### Symptoms

Participants described their first symptoms, the deformities they developed over time, and how they felt until they were diagnosed with leprosy. They mentioned functional limitations, such as “General weakness” (P7), or “There is no power in my hand, (…). The work also was difficult for me” (P10). When asked if they had experienced a loss of sensation before, one participant replied: “Without knowing, I would hold the boiling vessel and get burns, and that’s how I lost my fingers. Maybe I did not have a clear distinct of that, loss of sensation” (P11).

Participants also discussed side effects of the leprosy medication, with one recalling, “When I used to consume MDT, I used to get vomiting and all” (P7).

Most participants mentioned visible body changes, particularly ulcers, as one of the most prominent and limiting symptoms. As one stated, “Repeatedly, I am getting the ulcer. (…) they removed my foot, because bone was there (…) and bone was infected” (P12).

#### Restrictions

Participants described their restrictions due to their disabilities and the drastic effects on their daily lives. One woman explained, “I am not able to do any work or daily activities, I am away from all daily activities, not able to hold any objects. I am all dependent” (P6). Another recalled, “Agricultural work I used to do, but once I had problems in the foot I had to stop” (P12). A further women affected by leprosy reflected, “I cannot dress myself properly, that is the problem. And myself, even hair, even tight only, not possible” (P4).

Participants were forced to interrupt or stop their education, having a long-term impact on their prospects. One participant recounted “After this, due to health problems, I never continued education, I stopped” (P6). Another described, “I used to work in a school, I used to sweep the floors and things like that, but now, I had to stop, I am not working, I am begging.” (P15)

#### Emotional state

The symptoms evoked different reactions in the women: Some were anxious and worried, whereas others did not care about their symptoms that much, as long as they were not experiencing pain or restrictions on actions in their everyday lives. One participant recalled, “By seeing the patches, I was a lot afraid” (P6). Another said, “We were thinking (…) it would go away on its own. We did not think it is leprosy” (P16).

One participant reflected on her relationship and her feelings towards her husband because she was no longer able to do the household chores as before due to her disability: “I am imperfect. (…) I feel guilty” (P4).

After their diagnosis, participants felt distressed and saddened by their circumstances. They described the emotional impact of learning they had leprosy and the resulting change in how others treated them. Some reacted negatively, losing hope. One participants recounted, “Actually, when I realised that I was having leprosy, I used to feel very upset. It was very hard for me to accept it, because before that I did not accept it, did not care about it. (…) But after my husband’s family started separating me from the whole group, that was the time I started getting upset” (P15). Another expressed a similar feeling: “I was very upset, I cried a lot, I cannot express the sadness I had at that time, I cannot express in words” (P17). For some, the despair was even more severe, “Knowing what it is, I wanted to get suicide” (P7).

One woman described her reaction to other people’s behaviour when they made her eat her food in a different room: “That caused immense sadness, then I started crying and I started a big fight. (…) I was so hurt and angry and upset about what is happening and then also I thought why to live? People are treating me like this, they are separating me from everybody else, better to die” (P15). Alongside personal anguish, participants also expressed concerns for their future and that of their children. As one put it, “To get partner, I am feeling due to that disease. If they came to know that disease, then I am afraid for that. Sometimes people divorce due to leprosy” (P3). Another admitted, “I am scared that I will transport the disease to my son. So, I used to pray that my children should not get it” (P10).

### Theme 5: visits at healthcare facilities

#### Several institutions

Many participants visited multiple facilities, with the time before receiving a leprosy diagnosis ranging from a few days to several years. One participant recalled, “At least five hospitals I went to” (P13). Another noted “Same day we went and collected the medicine” (P6). For others, it took longer, one said: “One month” (P9).

The repeated visits to multiple medical facilities and the lack of symptom improvement caused participants to become increasingly worried about their health. One expressed, “I was worried (…), what is the disease, what is that? Just worried. Why is it happening? Getting pain and patches, so far, we have consulted many doctors, and I have taken many medicines. I just worried and used to cry. (…) Still, it is not subsided” (P6).

#### Journey to healthcare facilities

Participants encountered multiple challenges accessing healthcare, including distance, costs, time constraints, and the need for accompaniment, with their mobility further hindered by physical deformities. One participant shared, “My health condition was not good. I was not able to walk. My brother was able to lift and hold me and sit on the bike. He used to take me” (P7). For another, her disability and amputations led to the need to change their way of getting to a healthcare facility: “First, we used to come by bus, but recently, the last time we came by taxi” (P12). Many described public transport as burdensome and time-consuming. One woman explained, “I have difficulties with the bus travel because they will not stop where I say, they will stop at different places. (…) You have to be very dominant and jump” (P10), while also noting, that it takes her “3 hours to get here” (P10).

Another participant recounted her journey with her mother to the healthcare facility as follows: “Train, so we had to change three times. From my place to railway station, we had to go by bus. Again, from there, we had to catch the train, come here, again, we had to get on, again take another bus when I had to come here. So, it was difficult for us, especially as ladies, women, you know” (P16).

#### Treatment

Participants received various treatments for their leprosy, including naturopathic remedies, medications, MDT, physiotherapy, surgery, and special footwear. One woman recalled, “Some trees and leaves, in some villages they have given some medication” (P6). Another explained, “Right hand also, they did small skin graft, because already abduction of the fingers” (P7). A further participant described, “They gave treatment, treatment in the sense of medicine, injections, and rest” (P12).Some participants relied on external resources, like custom-made shoes, to manage their daily lives. As one participant put it, “I cannot walk without them” (P11). Some symptoms were only discovered at healthcare facilities, such as the physiotherapy department: “I never noticed that weakness is there. I never feel that this hand has become weak or something, I never felt it” (P1).

Eventually, all participants were referred to the SRH in Hyderabad by other healthcare workers, patients, or people familiar with the facility. Some, however, were referred only after their medication was finished. One woman explained, “A big problem was there, when I went to the first hospital, that time only weakness is there, no deformity. But they said, only after one-year treatment we will send you. Meanwhile, I got deformity” (P3).

After reconstructive surgery, participants reported high satisfaction with their care and treatment. One participant stated: “That time I am facing the pain and deformity, but now, there is no pain, no deformity, that’s why I am free, I feel happy” (P7).

#### Financial burden

Discussions on healthcare visits often highlighted the financial burden, especially with private facilities, making medical bills a significant challenge and a deciding factor for some in seeking help. One participant noted, “Some test they did, for that and medication and consultation I had to pay money” (P7). Another explained how costs influenced her healthcare access: “So, whenever I saved money, I used to go, otherwise I was not able to” (P10).

#### Lack of communication

Some participants were diagnosed with leprosy at healthcare facilities but were not informed about the disease, receiving treatment or referrals without understanding their condition. One woman recalled, “I completed MDT for one year, even though I never knew it is leprosy” (P6). Another explained, “So, when I got that boil, I went to the government hospital near my place, and then they gave this MDT leprosy medicine. But they did not tell me, that it is leprosy, so even at that time, I did not know that I have leprosy, but I was taking medicine” (P16).

In some cases, healthcare workers discussed the diagnosis only with family members or husbands, not directly informing the participants as patients. As one participant described: “P: They have diagnosed, but they never told me. Only my husband knows. (…) That doctor said to my husband and husband took medicine for one year. (…) He knows that I am suffering with leprosy, but he never tells me, that I am suffering with leprosy. But medication he has given me every month” (P4).

### Theme 6: individual factors

#### Care-seeking behaviour

One participant said that she did not go and see the doctor anymore after developing deformities because she thought that the medicine she got there did not help her: “Before this, I used to go, but now I am not going anymore. If I have some fever or anything; I, myself, will just go and get the medicine. (…) That medicine is not suiting me” (P12).

#### Lack of knowledge

An important aspect of each interview was the participants' lack of knowledge about leprosy before visiting a healthcare facility. Most women affected by leprosy stated they knew nothing about the disease before being diagnosed and educated. One participant admitted, “I have never seen any suffering leprosy patients; I don’t know anything about leprosy before diagnosis” (P8).

For one interviewee, the only association with leprosy came from seeing people in her village who went begging: “I did not know what is leprosy. When I saw the people walking and begging. And I have heard people talking, these are leprosy patients, so I have seen them” (P10).

Others lacked general medical knowledge. One participant, raised in a rural area without family support, explained, “I was very young at that time; I did not know that somebody called a doctor is there” (P15).

#### Lack of trust in medical advice

Individual attitudes towards the disease, treatment options, and healthcare workers' advice were often dismissive, leading to poor treatment adherence. Some participants expressed a sense resignition and felt they could do nothing to change their situation, as one remarked, “It is just fate” (P20). Others openly admitted ignoring medical instructions. One woman recalled, “Then they told me, better stop that medicine, the Prednisolone tablets. But I continued, even though the doctor said, better to stop” (P6). Another confessed, “I would go and as soon as they would bandage me, I would go and put it in the water. So, that is why, actually the treatment also did not go according to plan. Medicine also, I would just show that I am swallowing but then spit it out” (P15).

### Awareness gap

One of the biggest hurdles for participants was recognising symptoms and the need for urgent help, with some overlooking the loss of sensation due to focusing on basic needs. One participant recounted, “While cooking, I was developing blisters and ulcers, but I did not realise that it is loss of sensation, and I did not know the disease” (P14).

## Discussion

In summary, the participating women depended significantly on their social surroundings, particularly on their husbands or parents, for their individual healthcare decisions. Abandonment, as well as fear of discrimination often delayed the necessary treatment and led to a worsening of the health status. This is in line with previous studies on factors delaying leprosy diagnosis in women [16, 27]. Our study in particular highlights women’s dependence on others for healthcare, poverty, and societal rejection due to deformities [16]. To illustrate the complex interaction between individual experiences and systematic barriers, Levesque et al.’s Framework was used. This enabled us to portray different levels of barriers from the perceptions of symptoms until the usage of healthcare services for women affected by leprosy with G2D [26]. The study demonstrates how structural barriers, such as limited access to healthcare, and emotional barriers, like fear and isolation, intersect and result in delayed care-seeking among women with leprosy and G2D.

The participants identified themselves with strict gender roles, as married women and mothers and prioritised housework and childcare over their health, leading to injuries, delayed care, and the progression of leprosy. Even though another study did not specifically look at leprosy or other chronic illnesses, their study on the social status of women in India is still relevant in this context [14]. In a patriarchal society like India, where gender inequality is deeply rooted, discrimination against women happens on many levels, including education, employment and healthcare [14]. This issue transferred to women dealing with leprosy makes clear that the participants had to face a double burden of their disease and their disadvantaged social role as women in India [14].

The stigma associated with leprosy is deeply rooted in the Indian society [29]. Traditions, customs, and social patterns are hard to change [30]. In this study, in particular, women living in rural areas were affected by strictly enforced cultural norms. They associated leprosy with poverty, begging, deformities, and superstition. It was also related to sorcery and witchcraft.

This study revealed stigmatisation among leprosy patients and highlighted the lack of research specifically examining stigma among leprosy patients themselves, despite the existence of broader studies on stigma and leprosy [31]. A study from Nepal that examined the mental health problems of leprosy patients observed that leprosy leads to self-stigmatisation, low self-esteem, depression, and negative thinking in patients [32]. The participants likely experienced similar mental health effects from leprosy, projecting their negative self-perceptions onto other patients.

Participants although not personally mistreated were still preoccupied with their disease and physical changes, developing avoidant behaviour, including delaying medical help. This aligns with other studies showing that women in India who internalise negative beliefs tend to wait longer to seek care [16], as observed in our study, with up to several years until receiving the final leprosy diagnosis.

Reactions to symptoms in this study ranged from indifference to fear, with some participants, especially in rural areas, not recognising the need for concern. Lack of awareness about the disease and symptoms led to a carefree attitude, delaying diagnosis and treatment. Most participants sought help only when their symptoms began to cause restrictions. This aligns with another study made in the southern part of India where newly diagnosed, still untreated patients delayed treatment due to unawareness of symptoms [33]. Improving healthcare-seeking behaviours alongside increasing leprosy awareness could encourage more people, especially women, to report early symptoms at healthcare facilities.

In addition, disabilities themselves were a barrier for participants to access healthcare services. Participants from this study mentioned the challenges they had to overcome using public transport to get to a healthcare facility. Some patients could not walk at that time, so they depended on others to get to a healthcare facility. This is in accordance with another study that described a correlation between women affected by leprosy being dependent on their surroundings and the delay in seeking help [16].

The symptoms of leprosy and the diagnosis had a strong influence on the emotional state of the women during the process until they were diagnosed and treated. A study from Brazil showed that patients who are afraid of being excluded from their community are ten times more likely to delay seeing a doctor for their leprosy symptoms [34]. Despite differing geographical and cultural contexts, India and Brazil share parallels in their leprosy situations, with regional disparities and social inequalities placing a heavier burden on poorer classes, making leprosy a persistent public health challenge in both countries [34]. This indicates that women in India and Brazil face barriers to timely leprosy diagnosis and fear social exclusion.

Visiting several institutions and numerous hospitals, which often resulted in several days without work or income to buy food, contributed to the progression of leprosy. These findings mirrored those of another study that examined individual and community factors contributing to delays in leprosy diagnosis among patients with G2D from twelve countries, including India [35]. Initial visits to traditional or private medical providers delayed leprosy diagnosis [35]. This aligns with this study, where all patients received free leprosy treatment at government or affiliated institutions, suggesting global patterns contributing to delayed diagnoses.

A key finding of this study was that poor communication significantly contributed to deformities and disabilities in female leprosy patients. Lack of information at healthcare facilities left many unaware of the disease, preventing them from taking steps to prevent disabilities. Similar results were found in another Indian study, where less than half of the participants knew they had leprosy after diagnosis and MDT treatment at a healthcare facility [36].

Previous research in the same area has shown that missing treatment adherence is, next to the delay of diagnosis, another challenge in combatting the fight against leprosy [37]. The factors of communication and treatment adherence appeared interlinked, but further research is needed to understand this phenomenon.

Individual factors, such as care-seeking behaviour and lack of knowledge and awareness about leprosy, were crucial contributors to disability progression, supported by other studies, including a literature review and a mixed-methods study in India [16, 36].

The lack of trust in medical advice was another key factor in leprosy progression. One participant, for example, avoided seeing a doctor during pregnancy despite impairing symptoms, fearing the medication might harm her pregnancy. A study conducted in Brazil assessed how personal, external, clinical, and relational factors affected their management of the disease [38]. One main issue was inadequate patient knowledge and low trust in medical therapies, which led to poor disease outcomes [38]. A study from Costa Rica suggested considering patients’ perspectives about leprosy when talking about the diagnoses to improve their knowledge, treatment adherence and build a relationship of trust [39]. This underlines that education must be provided in a culturally sensitive way.

### Strengths and weaknesses

One key strength of the study was the diversity of female participants in terms of age, education, and location, which led to a more well-rounded understanding of women’s issues. Focusing solely on women with G2D allowed for an in-depth exploration of this under-researched group. The interviews yielded rich, detailed data, with many of the 20 participants sharing personal stories, often beyond the scope of the questions. Some noted they had never been asked so much about themselves and appreciated the opportunity to share, which helped them reflect on their experiences. The study gained credibility through in-person interviews, providing deep insights and ensuring reliability with thorough data coding. Additionally, the involvement of a researcher from a different cultural background added a valuable cross-cultural perspective, bringing fresh insights and uncovering new connections related to treatment adherence, communication and trust in medical advice.

The study had several methodological limitations, including potential biases. One significant limitation was selection bias, as all participants were recruited from the SRH clinic in Hyderabad. The women needed to be able to travel to the clinic independently, which possibly provided an advantage for women who were not as restricted in their travel abilities. Although they came from various regions, they were all referred to this clinic, which may not reflect the broader population of women with leprosy in Telangana. Furthermore, the regional limitation and the focus on one gender does not allow for much comparison, but rather an exploratory investigation into the relevant barriers of one neglected group of persons affected. Another limitation was the language barrier, as communication relied on a translator. While the translator was an experienced health worker, known and trusted by the participants, there remains a risk of miscommunication and misinterpretation. To minimise this, CN rephrased questions when necessary to ensure clarity and discussed the importance of accurate translation with the translator prior to the data collection. Cultural differences might have also affected participant engagement, and one participant’s speech difficulties, likely due to isolation, further complicated communication. Lastly, the study wasn’t intended to represent all women with leprosy in India but aimed to provide deeper insights into individual experiences.

### Recommendations for research and practice

This study underscores the importance of early diagnosis, treatment adherence, and a trusting relationship between female leprosy patients and healthcare workers. To reduce the burden of leprosy, further research is needed, involving the examination of more patients and additional regions. This is particularly necessary in India, where case numbers are higher in some regions than others and where diverse cultural backgrounds, patient behaviours, and healthcare facilities can influence disease outcomes [11, 14]. Men also need to be studied in greater detail to understand their help-seeking challenges, compare their experiences with those of women, and ensure equitable improvements in care. Another area requiring further investigation is trust in medical advice. This study revealed a connection between healthcare worker relationships and patient treatment adherence, which is crucial for health and preventing deformities. The Indian Leprosy Roadmap emphasises this focus, aiming to raise leprosy awareness through effective communication [40]. Recognising how essential communication is, we must ensure that these key elements are not only acknowledged but also effectively implemented within India’s current healthcare frameworks. A shift in societal attitudes and a relaxation of India’s strict social structures, norms, and rules could positively impact women’s health. They should be able to identify more as independent individuals and become more concerned about their health. This shift would allow them to be less preoccupied with social expectations, which often focus more on their duties and children than on their health status. To support early diagnosis and treatment, women need to be supported and empowered to seek help on their own. Awareness of the symptoms in the population needs to be raised, and barriers like the dependence on their surroundings and the financial burden that often comes with visiting a healthcare facility need to be addressed. At healthcare facilities, medical staff have to communicate with patients and those who accompany them about treatment options and possible complications to achieve good compliance and ensure patients are coming for follow-ups and are taking precautions to avoid injuries. Therefore, educating healthcare providers about leprosy and the significance of good communication for a trusty relationship with patients should be a key element in leprosy programs. Practically, since the financial situation of persons affected will not likely improve soon, leprosy programs need to encompass outreach activities, such as active case finding paired with the aforementioned health promotion. To ensure compliance with such different active detection methods, community engagement is critically needed in modifying interventions and designing research. The study highlights the importance of actively including female perspectives in participatory actions.

## Conclusion

The burden of leprosy remains high in India and requires attention. The delay in diagnosing leprosy is a primary factor contributing to the increasing proportion of G2D cases. This was further explored in this study, particularly focusing on women with G2D. Multiple social, cultural, and structural factors influence women’s healthcare-seeking behaviour and limit access to a timely and correct diagnosis and option for treatment. Reducing the rate of G2D cases is essential for alleviating the overall burden of leprosy. This requires a multi-faceted approach, including increased research, policy, and practical applications.

Beyond studying other regions in India and broader demographic groups, including men, the health system must take direct responsibility for building trust and ensuring equitable access to care. This includes strengthening primary healthcare infrastructure, integrating leprosy screening into routine services, and implementing the National Strategic Plan for Leprosy. Knowledge of symptoms, treatment, and the need for early consultation should be promoted at individual and community levels. Structural measures, such as referral pathways and monitoring of diagnostic delays, must be strengthened to ensure timely intervention. The emphasis should be on improving healthcare workers’ communication skills and educating them about the vital role of effective communication in building trusting relationships with patients and achieving high treatment adherence.

Coordinated action will help lower G2D cases and advance the WHO strategy aligned with Sustainable Development Goal 3 to achieve “good health and well-being” [41] for everyone.

## Supplementary Information

Supplementary Material 1: Appendix 1. Interview Guide.

Supplementary Material 2: Appendix 2 – Consent form - English.

Supplementary Material 3: Appendix 3 – Consent form – Telugu.

## Data Availability

Availability of data and material: All important data are shared as anonymised quotes in this paper. Additional data required can be requested through the corresponding author with reasonable justification.

## Abbreviations

ASHA: Accredited social health activist
DAHW: German Leprosy and Tuberculosis Relief Association
G2D: Grade 2 disability
GLRA: German Leprosy Relief Association
MDT: Multidrug therapy
NTD: Neglected tropical disease
SRH: Sivananda Rehabilitation Home
WHO: World Health Organisation
