# Supplementary material for: Barriers to seeking healthcare services and contributing factors to grade 2 disability among women affected by leprosy in Telangana, India – a qualitative study
Source: Int J Equity Health. 2025 Sep 29;24:240. doi: 10.1186/s12939-025-02642-9 (PMC12482034; doi:10.1186/s12939-025-02642-9)
Supplement: Supplementary file 1 — Supplementary Material 1: Appendix 1. Interview Guide. [file 12939_2025_2642_MOESM1_ESM.docx]

Appendix 1: Interview Guide

**Research Title:** Barriers for women affected by leprosy with Grade 2 disability to seek healthcare services in Telangana, India – A qualitative study

**Main Researcher:** Charlotte Nehring

Contact: [c.nehring@student.maastrichtuniversity.nl](mailto:c.nehring@student.maastrichtuniversity.nl)

**Research Participants:** Women affected by leprosy with disability grade 2

Start with introducing myself: Charlotte from Germany, studying Global Health at Maastricht University

talk about the study: for my master’s and doctoral thesis, I will collect data from several patients at SRH, no one will be able to identify someone from the results, and no names or personal characteristics will be named

everything will be anonymized

you can stop the interview or take breaks anytime.

First, I will ask some general questions, and then I would like to know your experiences of your pathway to your leprosy diagnosis, please be as specific as possible and feel free to talk as much as you want, we have enough time, and I would like to know your personal story.

**Demographic Information**

- How old are you?
- Where do you live (urban/rural area)? (If you live at SRH, where do you used to live? How far from SRH in km?)
- What is your employment status (change after leprosy diagnosis)?
- What is your occupation (change after leprosy diagnosis)?
- What is your relationship/marital status (family situation: married/single/divorced/stable relationship; any changes after leprosy diagnosis)?
- What is your parental status (children/how many)? (relationship to them?)

**Questions about the time between leprosy symptoms and diagnosis in chronological order**

*Knowledge and Experience*

- What did you **know** about leprosy before you noticed any symptoms? (cause of the disease, treatment, and whether it’s curable?)
- Did you hear the word leprosy before?
- Can you please describe the first **symptoms** of leprosy you experienced? The first changes you noticed? (loss of sensation? Did your skin look different?)
- **When** did you first notice any changes?
- How did you **experience** these symptoms and what were your **feelings** when you noticed the symptoms? Did you think about leprosy at that time? Were you afraid? Scared? Ashamed? Were you worried? Did it affect your mood?
- What **additional symptoms** have developed over time? (leading to disability)
- How did you **feel** then, how did this change over time?

*Visit of health care facility*

- Could you please tell me about your **experiences of visiting healthcare facilities** with your symptoms?
- How did you **prepare** for the visits? how did you decide where and when to go? what other factors did you consider and how did you experience it?
- Who was involved in your **decision-making** to seek medical help? (How involved were you?)
- How would you describe your health-seeking behavior in general? (How often do you go and see a doctor in general, not for your leprosy disease)
- What **external factors** influenced your health-seeking behavior for your leprosy symptoms? Other factors? (People you talked to? financial barriers? Time? resources?)
- How **far** was the healthcare institution from your home? (Is that a challenge?)
- How did you travel to the healthcare facility?
- Did someone accompany you? If yes, who came with you?
- Did you have to pay for anything?
- How often do you need to visit a healthcare facility?
- What other factors led to delaying your leprosy diagnosis (and treatment)?
- How do you feel about it?
- What **barriers** do you face to seeking healthcare services in general?

*Diagnose*

- How many healthcare facilities did you visit before you got diagnosed with leprosy?
- Can you please tell me **when** and **where** you got **diagnosed** with leprosy?
- Can you please **describe the situation** and how it made you feel?

*Social-cultural factors*

- How is leprosy seen in your community and society?
- Do you feel ashamed or stigmatized by your leprosy disease? If so, can you please tell me more about it?
- Did you ever experience any situations where you were treated differently because of your deformities or your leprosy disease?
- Can you tell me about your perspective on your role within your relationship/family situation?
- How did your relationship with family & friends change before and after getting diagnosed with leprosy / development of defomities?

*Structural factors*

- - If you are currently receiving treatment or got treatment in the past, do you or did you face any challenges in getting the medicine and care and taking it regularly?
  - Did you ever interrupt your leprosy treatment? Why?
  - What has bee nthe financial impact / burden of your leprosy disease?

**Questions about needs and demands**

- - What support or resources do you think would have helped you to get diagnosed earlier? What do you think needs to change for women to get diagnosed with leprosy earlier?
  - What assistance and support do you currently need for your treatment / leprosy-induced impairments from your community and surroundings?
  - Additionally, do you have any expectations regarding your needs from institutions such as the Sivananda Rehabilitation Home?
  - What changes do you think are needed to better support women with leprosy?
