# Supplementary material for: Barriers to seeking healthcare services and contributing factors to grade 2 disability among women affected by leprosy in Telangana, India – a qualitative study
Source: Int J Equity Health. 2025 Sep 29;24:240. doi: 10.1186/s12939-025-02642-9 (PMC12482034; doi:10.1186/s12939-025-02642-9)
Supplement: Supplementary file 2 — Supplementary Material 2: Appendix 2 – Consent form - English. [file 12939_2025_2642_MOESM2_ESM.docx]

Appendix 2: Consent Form - English

**Participant information Sheet and Consent form – adjustments made based onthe template form of the Sivananda Rehabilitation Home**

INFORMED CONSENT: Adults 18 years and over —English

**TITLE OF STUDY**:

Barriers for women affected by leprosy with Grade 2 disability to seek healthcare services in Telangana, India

**INSTITUTIONS**: Sivananda Rehabilitation Home (SRH) in Hyderabad, Maastricht University, GLRA (German Leprosy Relief Association) India, DAHW (Deutsche Lepra- und Tuberkulosehilfe; German Leprosy- and Tuberculosis Association)

**PRINCIPAL INVESTIGATORS**:

Charlotte Nehring

**CO-INVESTIGATORS**:

Dr S. Ananth Reddy (Chief Medical Officer at Sivananda Rehabilitation Home)

**Dr.Anil Fastenau (DAHW)**

**Dr.Srilekha Penna**

**PARTICIPATION INFORMATION:** You are being asked to take part in a study being performed by Charlotte Nehring and Sivananda Rehabilitation Home (SRH). You will participate in an interview that lasts approximately 45 – 90 minutes with Charlotte Nehring and a translator present. Interviews are going to be audio-recorded in case you agree. It is very important that you understand the following general principles that apply to all participants in our studies:

1) Your participation is entirely voluntary.

2) You may withdraw from participation in this study or any part of the study at any time with no penalty, harm, or loss of access to treatment.

3) After you read about the study, please ask any questions that will allow you to understand the study more clearly.

**INTRODUCTION TO THE TOPIC**:

Leprosy is a neglected tropical disease and remains a global health challenge. In 2022, almost 60 % of all leprosy cases occurred in India. It is an infectious disease caused by Mycobacterium leprae and is likely transmitted from person to person by droplets from nose to mouth during prolonged contact. Symptoms often only begin after a long incubation time of several years and can manifest as hypopigmented or erythematous skin patches. Furthermore, nerve damage with loss of sensitivity and irreversible nerve damage leading to disability can occur. The disease is curable with multidrug therapy for either six or twelve months. The disease is curable, but existing disabilities cannot be reversed, and the nerve damage and disabilities remain and must be taken care of. The disability can be subdivided into grade 0, with no visible deformities, grade 1 with impaired sensation, and grade 2 with additional visible deformities. Those, next to the disease itself, are associated with stigma impacting the patients and their families’ lives. The rate of grade 2 disability is associated with late diagnosis and a high disease burden. This research will investigate the barriers that women affected by leprosy with grade 2 disability must face to seek healthcare services in Telangana, India. Organisations, such as the German Leprosy Relief Association (GLRA) and the Sivananda Rehabilitation Home (SRH) are important non-governmental actors in the battle against leprosy in India. They provide structural tools, diagnostics and treatment facilities. This research will take place in collaboration with the GLRA and SRH. Qualitative data collection will be done via one-on-one interviews. Understanding the experiences of women from the first symptoms appeared until their diagnosis including access to treatment is crucial. This study aims to identify gaps and demands for female leprosy patients, which need to be considered to reduce the burden of leprosy.

**Participant information and Consent form – adjustments made with the template form of the Sivananda Rehabilitation Home**

**MAIN RESEARCHER:**

**Charlotte Nehring**

**WHO CAN PARTICIPATE IN THE STUDY:**

Any consenting female adult who fulfils the inclusion criteria can be enrolled in the study. The inclusion criteria are female, over 18 years old, diagnosed with leprosy, and having disability grade 2.

**RISKS, AND DISCOMFORTS OF STUDY PARTICIPATION**:

For the study participation, you will be asked to talk about your medical history and your experiences in connection to your leprosy disease, which might be uncomfortable to talk about and will be treated as a very sensitive topic for every participant.

**DATA MANAGEMENT:**

All data collected throughout the research process will be handled carefully and stored safely. During the fieldwork and the main researcher’s time in India the consent sheets, the notes taken from the main researcher, and the interview transcripts will be handled by the main researcher with no access for other people. The audiotapes and interview scripts will be password-secured on a laptop for this time. Afterwards, it will be ensured that the data is not accessible to other people and the data will be stored for 10 years on facilities provided by the University of Maastricht.

**BENEFITS**:

The participation in the study contributes to relevant leprosy research in Telangana. By attending the interviews your leprosy and your general health condition will be monitored and you will be informed about the results. You will be offered treatment for leprosy for free independently of your study participation. Your transport to the clinic for study visits will be compensated upon if you provide the staff with proof of your costs. If we find any medical problem or infection other than leprosy, you will be referred with them to a health dispensary.

PROCEDURES TO BE FOLLOWED:

If you participate in this study, we will ask you to answer with the use of semi-structured interview questions. This approach entails a balance between incorporating a topic guide with key questions to steer the conversation while also offering participants the freedom to talk about their experiences openly and in-depth. If written consent is given,the Interviews are going to be audio-recorded. If there is no written consent for recording the interviews, only digital notes will be taken from the interviews. No one, except for the principal investigator will have access to the data and it will be password-secured and saved after the research is finished for 10 years. This needs to be done in order to interpret the results.

**CONFIDENTIALITY**:

Any information you share with us will be kept private. Nobody except the principal investigator will have access to the data, they will be password-secured on a private laptop in India and password-secured on an external hard drive or facilities provided by Maastricht University after the research is finished. The data will be kept for at least 10 years for the possibility to publish the results in a journal. In case there will be a publication with the data, the paper will be passed on to the Sivananda Rehabilitation Home, so whenever the participants go there again, they can read it. There will not be any type of medical testing; we will only talk to you and ask questions. Each study participant will be given a unique study number. We will use this number, not your name, on the samples. Reports that come from this study will be in summary form. No one will be able to identify you from study reports.

**VOLUNTARINESS**:

You can withdraw at any time from the study without any negative consequences. Your current relationship to the Sivananda Rehabilitation Home or your treatment plan will not change if you do not want to take part in the study. If you would like to withdraw from the study, please contact Dr. S. Ananth Reddy (SRH study site PI) at (TELEPHONE NUMBER +91 9642869664). With or without participation in the study, you can seek care for leprosy infections at the health centre. If you have a leprosy infection, you can be treated for it by trained people from SRH. The treatments are free of cost.

**WHO TO CONTACT**:

If you have any questions about the research at any time or if you feel that you have been harmed by taking part in this study, you can contact Dr. S. Ananth Reddy (SRH study site PI) at (TELEPHONE NUMBER +91 9642869664) or contact Charlotte Nehring via email to c.nehring@student.maastricht@university.nl.

**Participant information and Consent form – adjustments made with the template form of the Sivananda Rehabilitation Home**

**STATEMENT OF PERMISSION:**

By signing this form of consent, I agree that I understand the aim and objectives of this study, that all questions I have regarding this study are answered and I am willing to participate in an interview for this study. Please sign or provide a thumbprint in the signature boxes if you agree to participate in the study.

Thank you for taking part in this study.

Participant’s Name

Place and Date

Participant’s signature or thumbprint

Person Obtaining Consent

Witness

**Audio Taping Consent**

Do you agree with audio recording the interviews for better analysis and research purposes?

Yes No

Place and Date Participant’s signature/thumbprint

____________________________________ ____________________________________
