# Supplementary material for: Barriers to seeking healthcare services and contributing factors to grade 2 disability among women affected by leprosy in Telangana, India – a qualitative study
Source: Int J Equity Health. 2025 Sep 29;24:240. doi: 10.1186/s12939-025-02642-9 (PMC12482034; doi:10.1186/s12939-025-02642-9)
Supplement: Supplementary file 3 — Supplementary Material 3: Appendix 3 – Consent form – Telugu. [file 12939_2025_2642_MOESM3_ESM.docx]

Appendix 3: Consent Form Telugu

**భాగస్వామ్య సమాచార పత్రిక మరియు సమ్మతి ఫారం**

**ప్రజ్ఞాసమ్మతి:** 18 సంవత్సరాల మరియు పై వయస్సు ఉన్నవయోజనులు — తెలుగు

**అధ్యయనశీర్షిక:**

భారతదేశంలోని తెలంగాణలో ఆరోగ్య సంరక్షణ సేవలను పొందేందుకు గ్రేడ్ 2 వైకల్యంతో కుష్టు వ్యాధితో బాధపడుతున్న మహిళలకు అడ్డంకులు

**సంస్థలు**: హైదరాబాద్‌లోని శివానంద పునరావాస గృహం (SRH), మాస్ట్రిక్ట్విశ్వవిద్యాలయం, GLRA (జర్మన్లెప్రసీరిలీఫ్అసోసియేషన్) భారతదేశం, DAHW (Deutsche Lepra-und Tuberkulosehilfe; German Leprosy- and TuberculosisAssociation)

**ప్రిన్సిపల్ఇన్వెస్టిగేటర్లు:**

షార్లెట్నెహ్రింగ్ (మాస్ట్రిక్ట్యూనివర్సిటీలోగ్లోబల్హెల్త్స్టూడెంట్)

**సహపరిశోధకులు:**

డాక్టర్ఎస్. అనంత్రెడ్డి (శివానందపునరావాసగృహంలోచీఫ్మెడికల్ఆఫీసర్)

డాక్టర్అనిల్ఫాస్టెనో (DAHW)

డాక్టర్శ్రీలేఖపెన్నా

**పాల్గొనే సమాచారం :** షార్లెట్నెహ్రింగ్మరియు శివానంద పునరావాసగృహం (SRH) నిర్వహిస్తున్నఅధ్యయనంలోపాల్గొనవలసిందిగా మిమ్మల్నిఅడుగుతున్నారు. మీరుషార్లెట్నెహ్రింగ్మరియు అనువాదకుడితో సుమారు 45 - 90 నిమిషాల పాటు జరిగే ఇంటర్వ్యూలో పాల్గొంటారు. మీరు అంగీకరిస్తే ఇంటర్వ్యూలు ఆడియో-రికార్డ్చేయబడతాయి.

మాఅధ్యయనాలలోపాల్గొనేవారందరికీవర్తించేక్రిందిసాధారణసూత్రాలనుమీరుఅర్థంచేసుకోవడంచాలాముఖ్యం:

1) మీభాగస్వామ్యంపూర్తిగాస్వచ్ఛందంగాఉంటుంది.

2) ఎలాంటిజరిమానా, హాని లేదా చికిత్సకు ప్రాప్యతను కోల్పోకుండా మీరు ఎప్పుడైనా ఈ అధ్యయనంలో లేదా అధ్యయనంలో ఏదైనా భాగం నుండి ఉపసంహరించుకోవచ్చు.

3) మీరు అధ్యయనం గురించి చదివిన తర్వాత, దయచేసి అధ్యయనాన్ని మరింత స్పష్టంగా అర్థంచేసుకోవడానికి మిమ్మల్ని అనుమతించే ఏవైనా ప్రశ్నలు అడగండి.

**అంశానికి పరిచయం :**

లెప్రసీ అనేది నిర్లక్ష్యం చేయబడిన ఉష్ణ మండల వ్యాధి మరియు ప్రపంచ ఆరోగ్య సవాలుగా మిగిలి పోయింది. 2022లో, దాదాపు 60% లెప్రసీ కేసులు భారతదేశంలోనే సంభవించాయి. ఇది మైకోబాక్టీరియం లెప్రే వలన సంక్రమించే ఒక అంటువ్యాధి మరియు దీర్ఘకాలంకలిపే సమయంలోముక్కు నుండి నోటికి బిందువుల ద్వారా వ్యక్తి నుండి వ్యక్తికి సంక్రమించే అవకాశం ఉంది. లక్షణాలు తరచుగా అనేక సంవత్సరాల సుదీర్ఘపొదిగే సమయం తర్వాత మాత్రమే ప్రారంభమవుతాయి మరియు హైపోపిగ్మెంటెడ్లే దాఎరిథెమాటస్స్కిన్ప్యాచ్‌లుగా వ్యక్తమవుతాయి.ఇంకా, సున్నితత్వంకోల్పోవడంతోనరాల నష్టం మరియు వైకల్యానికి దారితీసే కోలుకోలేని నరాలనష్టం సంభవించవచ్చు. ఆరు లేదా పన్నెండు నెలలపాటు మల్టీడ్రగ్థెరపీతో వ్యాధినయమవుతుంది. వ్యాధినయమవుతుంది, కానీ ఇప్పటికే ఉన్న వైకల్యాలను తిప్పికొట్టడం సాధ్యంకాదు మరియు నరాల నష్టం మరియు వైకల్యాలు అలాగే ఉంటాయి మరియు జాగ్రత్త తీసుకోవాలి. వైకల్యాన్ని గ్రేడ్ 0 గా విభజించవచ్చు, కనిపించే వైకల్యాలు లేకుండా, గ్రేడ్ 1 బలహీనమైన సంచలనంతో మరియుగ్రేడ్ 2 అదనపు కనిపించేవైకల్యాలతో. వ్యాధి ప్రక్కన ఉన్నవారు, రోగులు మరియు వారికుటుంబాల జీవితాలను ప్రభావితం చేసే కళంకంతో సంబంధం కలిగిఉంటారు. గ్రేడ్ 2 వైకల్యం రేటు ఆలస్యంగా నిర్ధారణ మరియు అధిక వ్యాధిభారంతో సంబంధం కలిగి ఉంటుంది. ఈ పరిశోధన భారతదేశంలోని తెలంగాణలోని గ్రేడ్ 2 వైకల్యంతో కుష్టువ్యాధితో బాధపడుతున్న మహిళలు ఆరోగ్య సంరక్షణ సేవలను పొందేందుకు ఎదుర్కొనే అడ్డంకులను పరిశీలిస్తుంది. జర్మన్లెప్రసీరిలీఫ్అసోసియేషన్ (GLRA) మరియు శివానందపునరావాసగృహం (SRH) వంటి సంస్థలు భారతదేశంలో కుష్టువ్యాధికి వ్యతిరేకంగా పోరాటంలో ముఖ్యమైన ప్రభుత్వేతర పాత్రధారులు.వారు నిర్మాణ సాధనాలు, రోగ నిర్ధారణ మరియు చికిత్స సౌకర్యాలను అందిస్తారు. ఈ పరిశోధన GLRA మరియు SRH సహకారంతో జరుగుతుంది. ఒకరిపై ఒకరు ఇంటర్వ్యూల ద్వారా గుణాత్మక డేటాసేకరణ జరుగుతుంది. మొదటి లక్షణాలు కనిపించినప్పటి నుండి వారిరోగనిర్ధారణ వరకు చికిత్సకు ప్రాప్యతతో సహా మహిళల అనుభవాలను అర్థంచేసుకోవడం చాలా ముఖ్యమైనది. ఈ అధ్యయనం స్త్రీ కుష్టురోగుల కోసం ఖాళీలు మరియు డిమాండ్‌లను గుర్తించడం లక్ష్యంగా పెట్టుకుంది, ఇది కుష్టువ్యాధి భారాన్ని తగ్గించడానికి పరిగణించాల్సిన అవసరం ఉంది.

**పాల్గొనే వారి సమాచారం మరియు సమ్మతిఫారమ్**

**ప్రధానపరిశోధకుడు:**

షార్లెట్నెహ్రింగ్: మాస్ట్రిక్ట్విశ్వవిద్యాలయం నుండి గ్లోబల్హెల్త్స్టూడెంట్యొక్కమాస్టర్.

**అధ్యయనంలో ఎవరు పాల్గొన గలరు:**

చేరిక ప్రమాణాలను నెరవేర్చే ఎవరైనా సమ్మతించే మహిళా పెద్దలు అధ్యయనంలో నమోదు చేసుకోవచ్చు. చేరిక ప్రమాణాలు స్త్రీలు, 18 ఏళ్లుపై బడిన వారు, కుష్టువ్యాధితో బాధ పడుతున్నారు మరియు వైకల్యం గ్రేడ్ 2 కలిగి ఉన్నారు.

**అధ్యయనంలో పాల్గొనే ప్రమాదాలు మరియు అసౌకర్యాలు:**

అధ్యయనంలో పాల్గొనడంకోసం, మీరు మీ వైద్య చరిత్ర మరియు మీ లెప్రసీవ్యాధికి సంబంధించి మీ అనుభవాల గురించి మాట్లాడమని అడగబడతారు, ఇది మాట్లాడటానికి అసౌకర్యంగా ఉండవచ్చు మరియు ప్రతి పాల్గొనే వారికి చాలాసున్నితమైన అంశంగా పరిగణించ బడుతుంది.

**సమాచార నిర్వహణ:**

పరిశోధన ప్రక్రియలో సేకరించిన మొత్తం డేటా జాగ్రత్తగా నిర్వహించ బడుతుంది మరియు సురక్షితంగా నిల్వ

చేయబడుతుంది. ఫీల్డ్‌వర్క్సమయంలో మరియు భారతదేశంలో ప్రధాన పరిశోధకుడి సమయంలో సమ్మతి షీట్‌లు, ప్రధాన పరిశోధకుడి నుండి తీసుకున్న గమనికలు మరియు ఇంటర్వ్యూ ట్రాన్‌స్క్రిప్ట్‌లు ఇతర వ్యక్తులకు ప్రాప్యత లేకుండా ప్రధాన పరిశోధకుడిచే నిర్వహించ బడతాయి. ఈ సమయంలో ఆడియోటేప్‌లు మరియు ఇంటర్వ్యూస్క్రిప్ట్‌లు ల్యాప్‌టాప్‌లో పాస్‌వర్డ్-భద్రపరచ బడతాయి. ఆతర్వాత, డేటా ఇతర వ్యక్తులకు అందుబాటులో లేదని నిర్ధారించ బడుతుంది మరియు మాస్ట్రిక్ట్విశ్వవిద్యాలయం అందించిన సౌకర్యాలపై డేటా 10 సంవత్సరాల పాటు నిల్వ చేయబడుతుంది.

**లాభాలు:**

అధ్యయనం లో పాల్గొనడం వల్ల తెలంగాణలో సంబంధిత లెప్రసీ పరిశోధనలకు దోహద పడుతుంది. ఇంటర్వ్యూలకు హాజరు కావడం ద్వారా మీ కుష్టువ్యాధి మరియు మీ సాధారణ ఆరోగ్య పరిస్థితి పర్యవేక్షించ బడుతుంది మరియు ఫలితాల గురించి మీకు తెలియజేయ బడుతుంది. మీరు మీ అధ్యయనంలో పాల్గొనకుండా స్వతంత్రంగా కుష్టువ్యాధికి ఉచితంగా చికిత్స అందించబడుతుంది. స్టడీ సందర్శనలకోసం క్లినిక్‌కి మీ రవాణాఖర్చులకు సంబంధించిన రుజువును సిబ్బందికి అందజేస్తే పరిహారంచెల్లించ బడుతుంది. మేము కుష్టువ్యాధి కాకుండా ఏదైనా వైద్య సమస్య లేదా ఇన్‌ఫెక్షన్‌ని కనుగొంటే, మీరు వారితో పాటు ఆరోగ్యడిస్పెన్సరీకి పంపబడతారు.

అనుసరించాల్సిన విధానాలు:

మీరు ఈ అధ్యయనంలో పాల్గొంటే, సెమీస్ట్రక్చర్డ్ ఇంటర్వ్యూ ప్రశ్నలను ఉపయోగించి సమాధానమివ్వమని మేము మిమ్మల్ని అడుగుతాము. ఈ విధానం సంభాషణను నడిపించడానికి కీలకమైన ప్రశ్నలతో టాపిక్గైడ్‌ను చేర్చడం మధ్య సమతుల్యతను కలిగి ఉంటుంది, అలాగే పాల్గొనేవారికి వారి అనుభవాల గురించి బహిరంగంగా మరియు లోతుగా మాట్లాడేస్వేచ్ఛను అందిస్తుంది. వ్రాతపూర్వక సమ్మతి ఇస్తే, ఇంటర్వ్యూలు ఆడియో-రికార్డ్చేయబడతాయి. ఇంటర్వ్యూలను రికార్డ్చేయడానికి వ్రాత పూర్వక సమ్మతి లేకపోతే, ఇంటర్వ్యూలనుండి డిజిటల్నోట్స్మాత్రమే తీసుకో బడతాయి. ప్రిన్సిపల్ ఇన్వెస్టిగేటర్మినహా ఎవరూ డేటాకు యాక్సెస్‌ను కలిగి ఉండరు మరియు 10 సంవత్సరాల పాటు పరిశోధన పూర్తయిన తర్వాత పాస్‌వర్డ్-భద్రపరచబడి, సేవ్చేయ బడుతుంది. ఫలితాలను అర్థంచేసుకోవ డానికి ఇది అవసరం.

గోప్యత:

మీరు మాతో పంచుకునే ఏదైనా సమాచారం ప్రైవేట్‌గా ఉంచ బడుతుంది. ప్రిన్సిపల్ ఇన్వెస్టిగేటర్మినహా ఎవరికీ డేటాకు ప్రాప్యత ఉండదు, వారు భారతదేశంలోని ప్రైవేట్ల్యాప్‌టాప్‌లో పాస్‌వర్డ్-భద్రపరచ బడతారు మరియు పరిశోధన పూర్తయిన తర్వాత మాస్ట్రిక్ట్విశ్వ విద్యాలయం అందించే బాహ్యహార్డ్డ్రైవ్లేదా సౌకర్యాలపై పాస్‌వర్డ్-భద్రపరచ బడతారు. జర్నల్‌లో ఫలితాలను ప్రచురించే అవకాశం కోసం డేటా కనీసం 10 సంవత్సరాల పాటు ఉంచ బడుతుంది. ఒక వేళ డేటాతో కూడిన ప్రచురణ ఉంటే, కాగితం శివానందపునరావాస గృహానికి పంప బడుతుంది, కాబట్టి పాల్గొనే వారు మళ్లీ అక్కడికి వెళ్లినప్పుడు, వారు దానిని చదవగలరు. ఏరకమైన వైద్య పరీక్షలు ఉండవు; మేము మీతో మాత్రమే మాట్లాడతాము మరియు ప్రశ్నలు అడుగుతాము. ప్రతి అధ్యయనంలో పాల్గొనే వారికి ప్రత్యేక అధ్యయన సంఖ్య ఇవ్వబడుతుంది. మేము నమూనాలపై మీ పేరు కాకుండా ఈ నంబర్‌ని ఉపయోగిస్తాము. ఈ అధ్యయనం నుండి వచ్చే నివేదికలు సారాంశ రూపంలో ఉంటాయి. అధ్యయన నివేదికల నుండి మిమ్మల్ని ఎవరూ గుర్తించలేరు.

**స్వచ్ఛందత:**

ఎటువంటి ప్రతికూల పరిణామాలు లేకుండా మీరు ఎప్పుడైనా అధ్యయనం నుండి ఉపసంహరించుకోవచ్చు. మీరు అధ్యయనంలో పాల్గొన కూడదనుకుంటే శివానంద పునరావాస గృహానికి లేదా మీ చికిత్స ప్రణాళికతో మీ ప్రస్తుత సంబంధం మారదు. మీరు అధ్యయనం నుండి వైదొలగాలను కుంటే, దయచేసి డాక్టర్ S. అనంత్రెడ్డి (SRH అధ్యయన సైట్ PI)ని (TELEPHONE NUMBER +91 9642869664)లో సంప్రదించండి. అధ్యయనంలో పాల్గొన్నా లేదా లేకున్నా, మీరు ఆరోగ్య కేంద్రంలో కుష్టువ్యాధి ఇన్ఫెక్షన్ల కోసం సంరక్షణను పొందవచ్చు. మీకు లెప్రసీ ఇన్‌ఫెక్షన్ ఉన్నట్లయితే, మీరు SRH నుండి శిక్షణ పొందిన వ్యక్తుల ద్వారా దానికి చికిత్స చేయవచ్చు. చికిత్సలు ఉచితం.

**ఎవరు సంప్రదించాలి :**

మీకు ఎప్పుడైనా పరిశోధన గురించి ఏవైనా సందేహాలు ఉంటే లేదా ఈ అధ్యయనంలో పాల్గొనడం వల్ల మీకు హాని జరిగినట్లు భావిస్తే, మీరు (TELEPHONE NUMBER +91 9642869664)లో డాక్టర్ S. అనంత్రెడ్డి (SRH అధ్యయనసైట్ PI) ని సంప్రదించవచ్చులేదా [c.nehring@student.maastricht@university.nlకి](mailto:c.nehring@student.maastricht@university.nlకి) ఇ-మెయిల్ద్వారా షార్లెట్నెహ్రింగ్‌ని సంప్రదించండి.

**పాల్గొనే వారి సమాచారం మరియు సమ్మతి ఫారమ్**

**అనుమతి ప్రకటన :**

ఈ సమ్మతి రూపంలో సంతకం చేయడం ద్వారా, ఈ అధ్యయనం యొక్క లక్ష్యం మరియు లక్ష్యాలను నేను అర్థంచేసుకున్నానని, ఈ అధ్యయనానికి సంబంధించి నాకు ఉన్నఅన్ని ప్రశ్నలకు సమాధానాలు ఉన్నాయని మరియు ఈ అధ్యయనం కోసం ఇంటర్వ్యూలో పాల్గొనడానికి నేను సిద్ధంగా ఉన్నానని అంగీకరిస్తున్నాను. మీరు అధ్యయనంలో పాల్గొనడానికి అంగీకరిస్తే, దయచేసి సంతకం చేయండి లేదా సంతకం పెట్టెల్లో బొటన వేలు ముద్రను అందించండి.

ఈ అధ్యయనంలో పాల్గొన్నందుకు ధన్యవాదాలు.

__________________________________________________

పాల్గొనే వారి పేరు

__________________________________________________

స్థలం మరియు తేదీ

__________________________________________________

పాల్గొనే వారి సంతకం లేదా బొటన వేలు

__________________________________________________

వ్యక్తి సమ్మతిని పొందడం

__________________________________________________

సాక్షిఆడియో టేపింగ్ సమ్మతి

మెరుగైన విశ్లేషణ మరియు పరిశోధన ప్రయోజనాల కోసం ఇంటర్వ్యూలను ఆడియో రికార్డింగ్ చేయడానికి మీరు అంగీకరిస్తారా?

అవును లేదు

స్థలం మరియు తేదీ పాల్గొనేవారి సంతకం/బొటనవేలు ముద్ర

_____________________________ ____________________________________
